# Quantitative gait analysis of idiopathic normal pressure hydrocephalus using deep learning algorithms on monocular videos

**DOI:** 10.1038/s41598-021-90524-9

**Published:** 2021-06-11

**Authors:** Sungmoon Jeong, Hosang Yu, Jaechan Park, Kyunghun Kang

**Affiliations:** 1 Department of Medical Informatics, School of Medicine, Kyungpook National University, Daegu, South Korea; 2 Research Center for Artificial Intelligence in Medicine, Kyungpook National University Hospital, Daegu, South Korea; 3 Department of Neurosurgery, School of Medicine, Kyungpook National University, Daegu, South Korea; 4 Department of Neurology, School of Medicine, Kyungpook National University, Daegu, South Korea

**Keywords:** Dementia, Hydrocephalus

## Abstract

A vision-based gait analysis method using monocular videos was proposed to estimate temporo-spatial gait parameters by leveraging deep learning algorithms. This study aimed to validate vision-based gait analysis using GAITRite as the reference system and analyze relationships between Frontal Assessment Battery (FAB) scores and gait variability measured by vision-based gait analysis in idiopathic normal pressure hydrocephalus (INPH) patients. Gait data from 46 patients were simultaneously collected from the vision-based system utilizing deep learning algorithms and the GAITRite system. There was a strong correlation in 11 gait parameters between our vision-based gait analysis method and the GAITRite gait analysis system. Our results also demonstrated excellent agreement between the two measurement systems for all parameters except stride time variability after the cerebrospinal fluid tap test. Our data showed that stride time and stride length variability measured by the vision-based gait analysis system were correlated with FAB scores. Vision-based gait analysis utilizing deep learning algorithms can provide comparable data to GAITRite when assessing gait dysfunction in INPH. Frontal lobe functions may be associated with gait variability measurements using vision-based gait analysis for INPH patients.

## Introduction

Idiopathic normal pressure hydrocephalus (INPH) is a rare neurological disorder. Of 563 autopsies presenting dementia neuropathology, only 9 (1.6%) were suspected as INPH^1^. In spite of this low incidence, diagnosing INPH is important because it is a potentially treatable neurological disorder. It is an idiopathic adult-onset syndrome involving nonobstructive enlargement of cerebral ventricles, and it is known by its symptoms of cognitive impairment, gait disturbance, and urinary dysfunction. While INPH can present with any of these classic clinical symptoms in varying degrees, the most frequent and important INPH clinical feature is gait disturbance. When diagnosing INPH, many neurosurgical centers suggest using the cerebrospinal fluid tap test (CSFTT)^2,3^. In addition, the CSFTT has a high positive predictive value for successful shunt surgery^2,3^. Further, the CSFTT response has been seen as a strong predictor of shunt effectiveness in INPH patients and a valuable marker for understanding the progression of the disorder^2,3^.

The GAITRite gait analysis system uses a portable walkway that is embedded with pressure sensors detecting footfalls as the patient walks on the mat^4^. The software enables documenting a wide range of gait parameters, including stride length, step width, walking speed, cadence, and foot placement angles^4^. Its validity and reliability have been demonstrated in various studies^5,6^.

Video sensors provide a rich source of information that can be used for gait analysis^7^. Recent evidence indicates that the vision-based gait analysis using artificial intelligence algorithms can be used to validly assess stride dynamics during walking^8,9^. A study of Parkinson’s disease patients showed that there was a high correlation in certain gait parameters, such as gait cycle time, stance phase (% of gait cycle time), swing phase (% of gait cycle time), stride length, walking velocity, and cadence, measured by the vision-based gait analysis method and the GAITRite gait analysis system^10^. In a previous study using the Vicon motion capture system as a reference system, it was reported that the vision-based gait analysis system seemed to have sufficient accuracy in measuring stride-to-stride variation in stride length^9^. Further, in our recent study using the GAITRite gait analysis system as a reference system, a vision-based gait analysis method using monocular videos was proposed to properly estimate temporo-spatial gait parameters by leveraging deep learning algorithms^11^. Gait analysis in INPH patients is important for both determining the severity of INPH and evaluating the improvements provided by the treatment regimen^3^. The vision-based gait analysis system can provide clinicians with a low-cost, non-intrusive, and easy-to-use system for quantitative gait analysis^12^.

Previous studies have conjectured that there is a correlation between INPH and frontal lobe dysfunction. Concerning cerebral blood flow in INPH, most previous studies have revealed that frontal-dominant perfusion decreases or whole-brain perfusion decreases using single photon emission computed tomography or positron emission tomography^13,14^. And gait disorders in INPH are classically described as slow, magnetic, and wide-based, also known as frontal gait^15^. It has been hypothesized that INPH gait dysfunction may be caused by frontal lobe dysfunction^16^. Stride time and stride length variability are both parameters associated with the control of the rhythmic stepping^17,18^. Increased gait variability is the result of inconsistent stepping patterns and reduced postural control during gait^19^. One previous study reported that a deterioration in dynamic balance function may result in an increase in gait variability and an increased risk of falls in patients with INPH^19^. The Frontal Assessment Battery (FAB) has been proven to be a reliable and short bedside cognitive and behavioral test to assess frontal lobe functions^20^. In a prior study of INPH patients, we found that stride time and stride length variability measured by the GAITRite system were correlated with FAB score^21^.

In this study, we investigated gait performance utilizing a quantitative gait analysis before and after the CSFTT in INPH patients. The quantitative gait data were simultaneously collected from the vision-based gait analysis system using artificial intelligence algorithms for monocular videos and the GAITRite gait analysis system. The aims of the study were (1) to evaluate the concurrent validity of the vision-based gait analysis method for temporo-spatial gait measurement using the GAITRite as the reference system and (2) to determine whether there was any relationship between stride time and stride length variability measured by the vision-based gait analysis system and FAB scores in INPH patients.

## Methods

### Participants

Study participants were prospectively recruited from patients at the Center for Neurodegenerative Diseases of Kyungpook National University Chilgok Hospital, South Korea between August 2017 to September 2019. INPH diagnoses were made using criteria presented by Relkin et al.^22^. The inclusion criteria for study participants were set as follows: 6 months progression or longer of gait disturbance along with either cognition or urinary symptoms, > 40 years of age, and normal CSF opening pressure. Brain MRI showed ventricle expansion (Evans’ ratio > 0.3) for all study participants, with no CSF flow obstruction. Exclusion criteria were as follows: patients with a hospitalization history of a significant psychiatric disorder, stroke, recent history of extensive alcohol use, or history of metabolic, neurological, or neoplastic dysfunctions that could show dementia symptoms. No participant in the study showed evidence of intracerebral hemorrhage, meningitis, head trauma, or other potential cause of hydrocephalus. A lumbar tap removing 30–50 ml of CSF was done for each INPH patient.

This study protocol was approved by the Institutional Review Board of Kyungpook National University Chilgok Hospital. All methods and procedures were performed in accordance with relevant guidelines and regulations. All study participants gave informed and written consent for the study, including information related to clinical data and MRI. Each patient also consented to having a CSFTT.

### Assessing illness severity

Comprehensive clinical scales for each INPH patient in the study was determined in the following manner. Dementia severity and general cognition were evaluated with the Clinical Dementia Rating Scale (CDR) and K-MMSE^23,24^. The FAB was used to ascertain frontal lobe symptoms^20^. The total FAB score ranged from 0 to 18, with a higher score meaning better performance. The INPHGS was employed to determine symptom severity for cognitive impairment, gait disturbance, and urinary disturbance after an unstructured interview with patients and caregivers^25^. The score for each symptom ranged from 0 to 4. Grade 0 indicates normal; grade 1 indicates mild subjective symptoms but no objective disturbance; grades 2, 3, and 4 indicate mild, moderate, and severe disturbance, respectively. Gait assessment included performance results on the Timed Up and Go (TUG) test and 10 m walking test^25–27^. The TUG test measures the length of time it takes a patient sitting in a chair to stand up, walk forward 3 m, and return to a seated position. Gait disturbance features related to INPH were measured using the Gait Status Scale (GSS)^25^. This scale focuses on 8 factors related to gait disturbance: (1) wide base gait; (2) independence in walking; (3) postural stability; (4) lateral sway; (5) festinating gait; (6) petit-pas gait; (7) gait freezing; and (8) disturbed tandem walking. A total GSS score of the 8 items, ranging from 0 to 16, was determined for each patient. A higher score reflected greater symptom severity.

### Quantitative gait assessment

A 5.8-m-long pressure-sensitive carpet system (GAITRite, CIR System, Havertown, PA) with a sampling rate of 120 Hz was used to determine gait measurements. Spatio-temporal gait parameters related to this study were measured. All participants were told to walk barefoot at a reasonable and self-selected speed without the use of any walking aid. The process was repeated 4 times to obtain sufficient data for analysis. To prevent acceleration and deceleration effects, participants started walking 1 m before reaching the active area of the electronic walkway and completed their walk 1 m beyond it. All patients were given time to rest between walking trials when requested to avoid fatigue. Each patient had a researcher walking alongside as a safeguard. Spatiotemporal gait parameters were determined using the GAITRite system as follows: stride length, step width, gait velocity, cadence, toe in/out angle, stride time, stance phase (%), and swing phase (%). The coefficient of variation (CV) for stride time and stride length was calculated as follows: SD of parameter × 100/mean of the parameter. All INPH patients were analyzed before the CSFTT, 32 of which were analyzed again after CSF removal.

The gait data were simultaneously collected from the vision-based gait analysis system using a monocular camera and the GAITRite gait analysis system. Video recordings were obtained at the end of the electronic walkway. The camera was located 0.5 m above ground level and directly before the participant, a position that provided a clear view of both feet. Previously described methods were used to investigate gait performance utilizing deep learning algorithms for monocular videos^11^. Briefly, our vision-based gait analysis system followed a two-step framework, which first detected a human bounding box found in the patient localization process and then estimated gait parameter values within the box using a single person gait regression model. To solve the multi-person appearance issue, we conducted the patient localization step, which detected and found the patient from video with help from the off-the-shelf object detection methods YOLO v3^11^. And then we selected a region of interest in the video frames to train a convolution neural networks (CNN) model for a gait parameter regression on a single person. The architecture of the single person model was designed to follow the layer configuration of ResNet18^11^, and we inflated the 2D CNN structure to 3D to interpret motion that was present within space–time voxels. There are two different types of gait parameters called average-type and CV-type gait parameters. (1) Average-type gait parameters could be estimated by the proposed single-person model from monocular video sequencings directly but (2) CV-type gait parameters require finding the ratio of the standard deviation to the average value. When we train a CNN model to estimate average-type and CV-type parameters at the same time, inaccurate average values in an early phase of the training can excessively amplify errors for the corresponding CV values. Consequently, gradients used for the CNN weights update can explode and the model becomes difficult to converge. To solve this problem, we split the training phase into 2 stages and trained the mean gait parameters first and CV gait parameters later. First, we trained the model to estimate mean values, except for CV values, in a similar way of a previous work. Second, we built a separate CV value estimation model by concatenating features from the mean estimation model with the last CNN layer. Based on these approaches, the mean-related features in the 1st stage could increase the stability of the learning process for the 2nd stage. Finally, we implemented our model with PyTorch, and used 4 NVIDIA TITAN V GPUs to train the CNN model. We also used Dask frameworks for hyper-parameter optimizations in a parallel way^11^. The dataset used in this study was different from the one used in our previous study that developed our vision-based gait analysis system using deep learning algorithms for monocular videos, so there was no overlap in data between the two studies^11^.

### Statistical analyses

The levels of agreement between the vision-based gait analysis method and the GAITRite gait analysis system were assessed using Pearson correlation coefficients and intraclass correlation coefficients (ICCs) of the type (2, k), as reported previously^28,29^. Bland–Altman plots were created to provide a visual representation of heteroscedasticity by plotting individual subject differences between the two systems against the individual mean of the two systems^28^. ICC values were interpreted as > 0.75 being excellent, 0.40–0.75 as good, and < 0.40 as poor^30^.

Pearson’s correlations were employed to investigate the relationship between stride time and stride length variability measured by the vision-based gait analysis system and FAB scores in INPH patients. Statistical significance was set at *P* < 0.05.

## Results

Forty-six INPH patients (35 CSFTT responders and 11 CSFTT non-responders) constituted the final sample for analysis. Response to the CSFTT was defined in detail elsewhere^21^. Baseline clinical findings of the study cohort are shown in Table 1. The subjects were 75.0 ± 6.7 years old.

**Table 1:** Demographic data and clinical characteristics of INPH patients at baseline.

Characteristics	Baseline
Gender, male	20 (43.5)
Age (year)	75.0 ± 6.7
Education (year)	7.7 ± 4.4
Duration of symptoms (year)	3.1 ± 2.2
K-MMSE	21.1 ± 5.4
CDR (0:0.5:1:2:3)	0:32:10:3:1
**INPHGS**	
GS-Gait	1.0 ± 0.2
GS-Cogn	2.5 ± 0.6
GS-Urin	1.4 ± 1.1
TUG	17.4 ± 5.8
10-m walking test	15.9 ± 6.9
GSS	7.4 ± 1.4
FAB	10.1 ± 3.1
Drainage volume of CSF	38.5 ± 2.9
CSF opening pressure (cm H_2_O)	9.9 ± 2.6
Evans’ ratio	0.33 ± 0.02

Data were collected before the CSFTT. Values denote number (%) or mean ± standard deviation.

INPH, idiopathic normal pressure hydrocephalus; CSFTT, cerebrospinal fluid tap test; K-MMSE, Korean version of Mini-Mental State Examination; CDR, Clinical Dementia Rating Scale; INPHGS, Idiopathic Normal Pressure Hydrocephalus Grading Scale; GS-Gait, INPHGS for gait; GS-Cogn, INPHGS for cognition; GS-Urin, INPHGS for urinary function; TUG, Timed Up-and-Go test; GSS, Gait Status Scale; FAB, Frontal Assessment Battery.

### Concurrent validity

**Figure 1:**
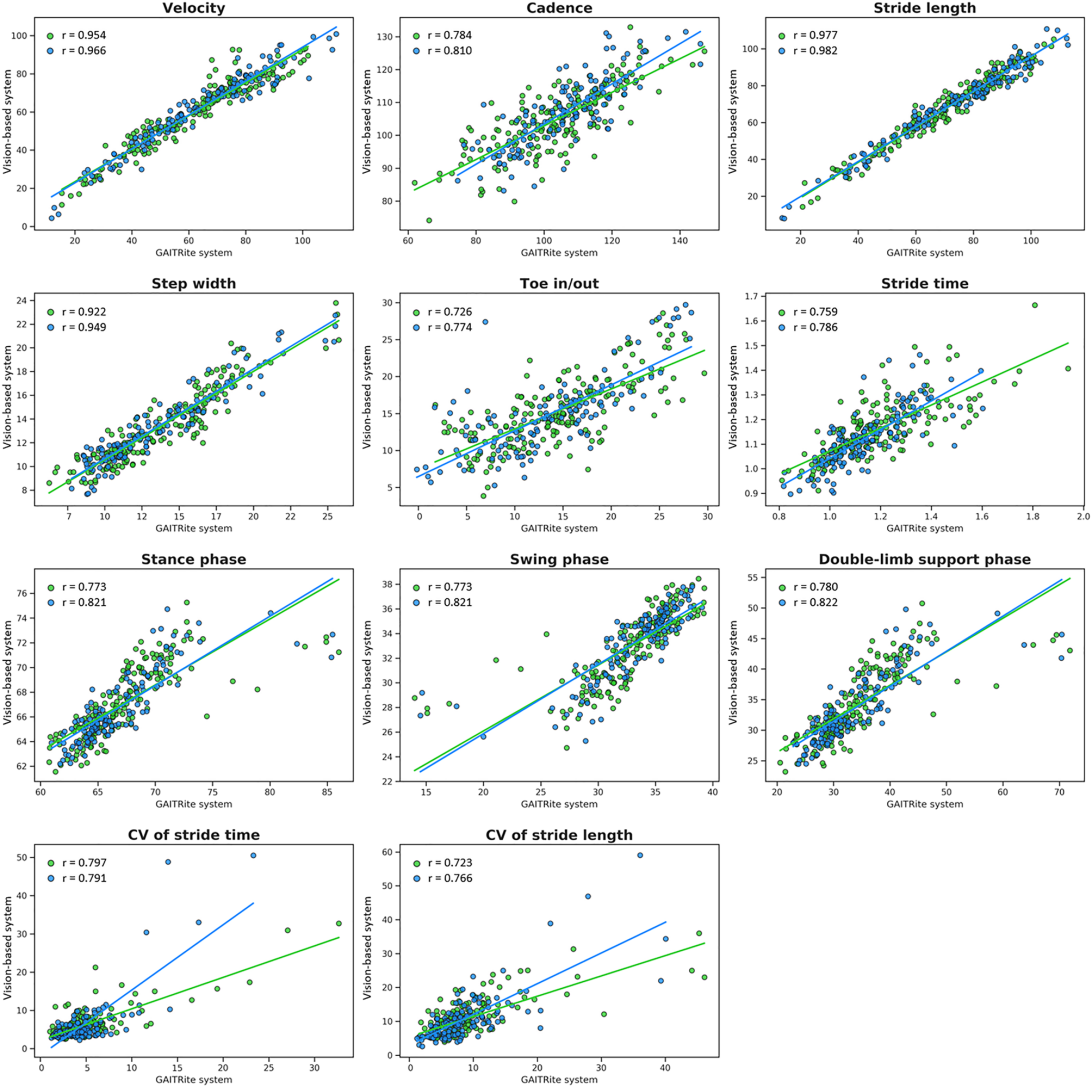
Relationships between the vision-based gait analysis system and the GAITRite gait analysis system for all parameters. Pearson’s correlation analyses assessing correlations between the two measurement systems for all parameters before and after the CSFTT. Green dots indicate data before the CSFTT while blue dots indicate data after the CSFTT. CSFTT, cerebrospinal fluid tap test; CV, coefficient of variability.

Before the CSFTT, significant correlations were detected between the vision-based gait analysis method and the GAITRite gait analysis system for all parameters (Table 2 and Fig. 1). There were very strong correlations between the two measurement systems for the gait velocity (r = 0.954; *P* < 0.001), stride length (r = 0.977; *P* < 0.001), and step width (r = 0.922; *P* < 0.001). There were strong correlations between the two measurement systems for the cadence (r = 0.784; *P* < 0.001), toe-out angle (r = 0.726; *P* < 0.001), stride time (r = 0.759; *P* < 0.001), stance phase (r = 0.773; *P* < 0.001), swing phase (r = 0.773; *P* < 0.001), double-limb support phase (r = 0.780; *P* < 0.001), CV of stride time (r = 0.797; *P* < 0.001), and CV of stride length (r = 0.723; *P* < 0.001). Before the CSFTT, the ICCs for all gait parameters were excellent (ranging from 0.805 to 0.982).

**Table 2:** Concurrent validity between the vision-based gait analysis system and the GAITRite gait analysis system for temporo-spatial gait parameters.

	Before CSF tap						24 h after tap					
	GAITRite system (mean ± SD)	Vision-based system (mean ± SD)	r	ICC	Mean difference	95% LoA	GAITRite system (mean ± SD)	Vision-based system (mean ± SD)	r	ICC	Mean difference	95% LoA
Velocity, cm/s	61.63 ± 20.29	59.47 ± 18.42	0.954	0.971	2.15	− 9.89 to 14.20	64.25 ± 21.80	62.21 ± 20.06	0.966	0.979	2.04	− 9.07 to 13.14
Cadence, steps/min	103.69 ± 15.49	104.73 ± 10.10	0.784	0.834	− 1.05	− 20.28 to 18.18	106.18 ± 14.26	107.19 ± 10.76	0.810	0.875	− 1.01	− 17.42 to 15.39
Stride length, cm	71.27 ± 19.18	68.35 ± 18.84	0.977	0.982	2.92	− 5.14 to 10.98	72.87 ± 21.69	70.08 ± 20.99	0.982	0.987	2.78	− 5.22 to 10.78
Step width, cm	13.65 ± 3.76	13.28 ± 3.04	0.922	0.945	0.37	− 2.60 to 3.34	13.62 ± 4.13	13.40 ± 3.29	0.949	0.960	0.22	− 2.62 to 3.05
Toe in/out, °	14.46 ± 6.31	15.33 ± 4.70	0.726	0.815	− 0.87	− 9.36 to 7.62	13.63 ± 6.79	14.93 ± 5.45	0.774	0.851	− 1.30	− 9.71 to 7.12
Stride time, s	1.18 ± 0.19	1.16 ± 0.12	0.759	0.808	0.03	− 0.23 to 0.28	1.15 ± 0.16	1.13 ± 0.12	0.786	0.849	0.02	− 0.17 to 0.21
Stance phase, %	67.16 ± 4.14	67.07 ± 2.86	0.773	0.840	0.09	− 5.09 to 5.27	66.77 ± 4.18	66.72 ± 2.86	0.821	0.868	0.06	− 4.73 to 4.85
Swing phase, %	32.84 ± 4.14	32.95 ± 2.86	0.773	0.840	− 0.11	− 5.28 to 5.07	33.23 ± 4.18	33.30 ± 2.86	0.821	0.868	− 0.08	− 4.87 to 4.72
Double-limb support phase, %	34.46 ± 8.20	34.30 ± 5.77	0.780	0.847	0.15	− 9.95 to 10.26	33.81 ± 8.23	33.61 ± 5.76	0.822	0.872	0.20	− 9.17 to 9.57
CV of stride time, %	4.77 ± 3.89	6.10 ± 4.02	0.797	0.860	− 1.33	− 6.26 to 3.60	4.68 ± 3.14	6.23 ± 6.77	0.791	0.735	− 1.55	-10.71 to 7.61
CV of stride length, %	8.62 ± 6.44	10.59 ± 5.34	0.723	0.805	− 1.97	− 10.78 to 6.83	8.76 ± 6.43	10.92 ± 7.61	0.766	0.839	− 2.16	− 11.78 to 7.47

CSF, cerebrospinal fluid; ICC, intraclass correlation coefficient; 95% LoA, 95% limits of agreement; CV, coefficient of variability.

Twenty-four hours after the CSFTT, significant correlations were detected between the vision-based gait analysis method and the GAITRite gait analysis system for all parameters (Table 2 and Fig. 1). There were very strong correlations between the two measurement systems for the gait velocity (r = 0.966; *P* < 0.001), stride length (r = 0.982; *P* < 0.001), and step width (r = 0.949; *P* < 0.001). There were strong correlations between the two measurement systems for the cadence (r = 0.810; *P* < 0.001), toe-out angle (r = 0.774; *P* < 0.001), stride time (r = 0.786; *P* < 0.001), stance phase (r = 0.821; *P* < 0.001), swing phase (r = 0.821; *P* < 0.001), double-limb support phase (r = 0.822; *P* < 0.001), CV of stride time (r = 0.791; *P* < 0.001), and CV of stride length (r = 0.766; *P* < 0.001). After the CSFTT, the ICCs for all gait parameters except CV of stride time were also excellent (ranging from 0.839 to 0.987).

**Figure 2:**
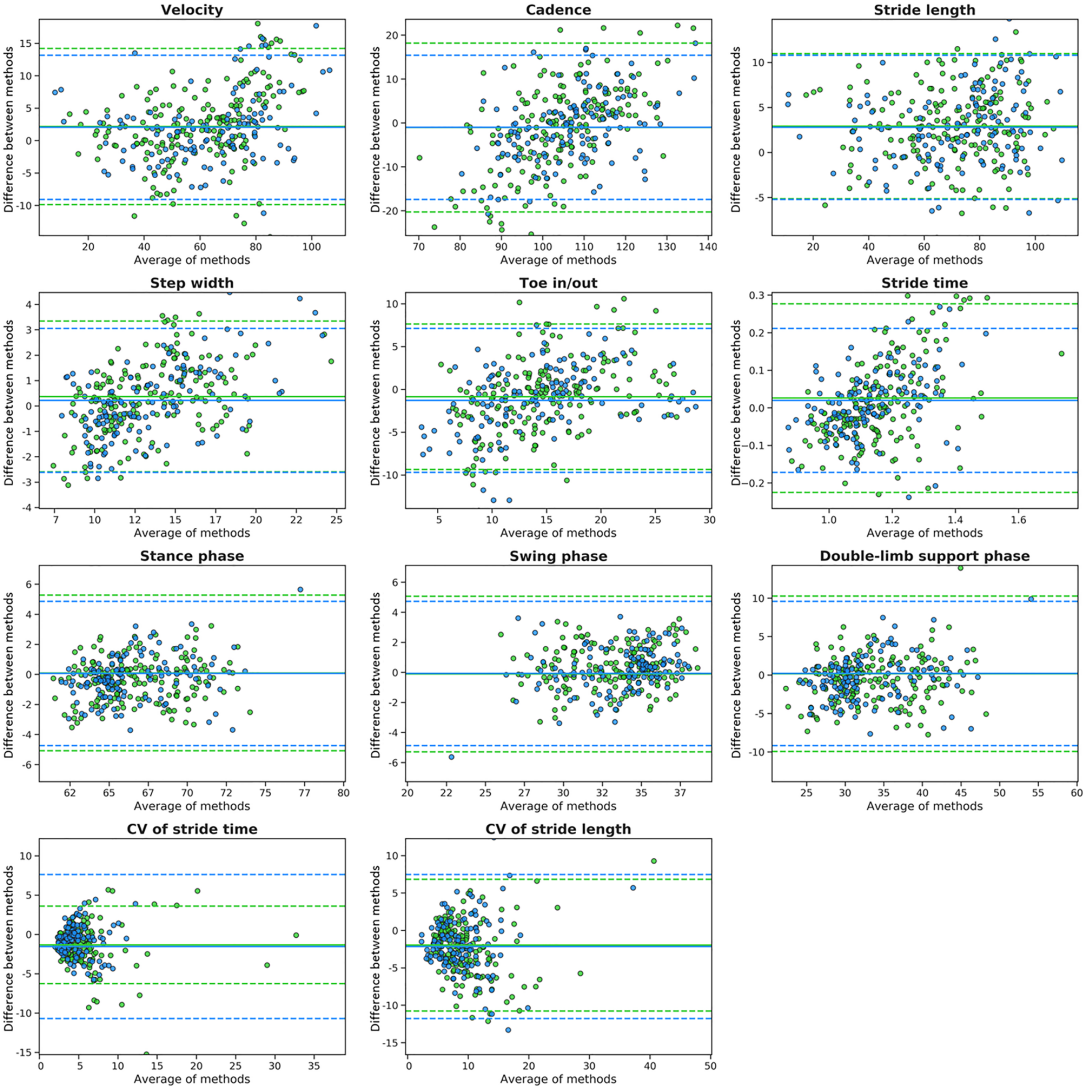
Agreements between the vision-based gait analysis system and the GAITRite gait analysis system for all parameters. Bland–Altman plot of all gait parameters measured by the two measurement systems before and after the CSFTT. Straight and dashed lines indicate mean differences and 95% limits of agreement, respectively. Green color indicates data before the CSFTT while blue color indicates data after the CSFTT. CSFTT, cerebrospinal fluid tap test; CV, coefficient of variability.

Figure 2 illustrates the Bland–Altman plot of all gait parameters measured by the vision-based gait analysis method and the GAITRite gait analysis system before and 24 h after the CSFTT. These plots showed that mean differences were almost all close to zero.

### Correlations between FAB Scores and gait variability measured by the vision-based gait analysis system in INPH

**Figure 3:**
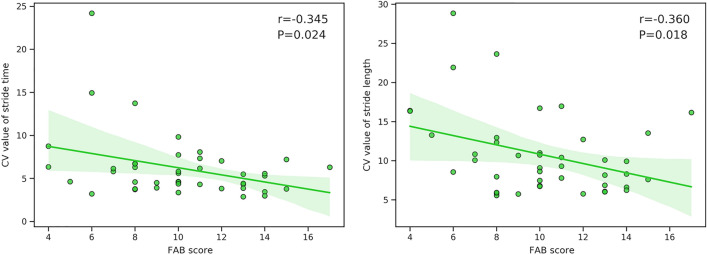
Scatterplots illustrating the relationships between FAB Scores and gait variability measured by the vision-based gait analysis system at baseline. Pearson’s correlation analyses assessing correlations between stride time and stride length variability and FAB scores at baseline. CV, coefficient of variability; FAB, Frontal Assessment Battery.

The FAB scores were negatively correlated with the CV value of stride time (r =  − 0.345; *P* = 0.024) and CV value of stride length (r =  − 0.360; *P* = 0.018) as measured by the vision-based gait analysis system (Fig. 3).

## Discussion

This study investigated the validity of a vision-based gait analysis system in INPH patients using artificial intelligence algorithms for monocular videos in comparison to a well-established gait analysis system. There was a strong correlation in 11 gait parameters between our vision-based gait analysis method and the GAITRite gait analysis system. Our results also demonstrated excellent agreement between the two measurement systems for all parameters (with the exception of CV of stride time after the CSFTT). Relative to its potential clinical application, we also showed that key gait parameters measured by the vision-based gait analysis system were correlated with the FAB score.

The excellent agreement between our vision-based gait analysis method and the GAITRite gait analysis system reveals several interesting points. First, we found that the vision-based gait analysis system was comparable to the GAITRite system for measuring stride time and stride length variability in INPH patients. Losing the ability to produce a steady gait rhythm, resulting in greater stride-to-stride variability, has been related to balance impairments that can lead to falls^31,32^. Increased stride-to-stride variability in stride time and stride length was significantly associated with a high risk for falling in older adults^17,33^. Falls are also clinically important in patients with INPH^34^. It was reported that more than half of INPH patients (56%) experienced falls^34^. Further, we previously reported that stride time and stride length variability were increased in INPH patients relative to controls^21^. Therefore, increased gait variability may be one of the main risk factors for falls in INPH patients^21^. We cautiously suggest that the vision-based gait analysis system has the potential to ‘bridge the gap’ between laboratory testing and clinical assessment of fall risk in INPH patients. Second, we found that the vision-based gait analysis system was also comparable to the GAITRite system for measuring gait velocity, stride length, step width, toe-out angle, stance phase, and double-limb support phase in INPH patients. INPH gait dysfunction has been traditionally characterized in a general way as lower-body parkinsonism^35^. However, according to recent studies using quantitative gait analysis instruments, INPH gait dysfunctions can be characterized more specifically as follows^21,34,36^. Dysfunctions possibly related to the basal ganglia circuitry may include decreased walking speed and short stride lengths^21,34,36^. And more importantly, dysfunctions possibly related to the cerebellar circuitry may include broad-based gait, outward rotation of the feet, increased stance phase, and increased double-limb support phase^21,34,36^. Both the step width and the foot angle have been standardly considered as balance-related gait parameters^36,37^. Phenomena such as enlarged step width and outward rotated feet can be interpreted as protective strategies to stabilize gait^36,37^. Cerebellar circuits are well established to be involved in controlling balance^38^. It has also suggested that hydrocephalus may directly compress and therefore distrupt frontopontocerebellar fibers as they descend toward the lateral ventricle^39^. In addition, a significant reduction in mean cerebral blood flow of the cerebellum was found for INPH patients compared with controls^40^. It has been hypothesized that the stance phase and double-limb support are stabilizers during normal gait in the elderly^41^. We also suggest that these quantitative and clinical methods may be used interchangeably when evaluating INPH patients. Third, after the CSFTT, our results also showed strong correlations and excellent agreement between the two measurement systems for all parameters except CV of stride time. The CSFTT is often thought of as an acute treatment for INPH^37^. Taking 30–50 ml CSF from the lumbar CSF space, as in the CSFTT, may create a situation identical to a ventricular shunt operation for a certain time period^2^. Further, changes in brain state can occur in INPH patients after CSF removal. For example, it was reported that CSF removal resulted in a change of the brain volume rather than only a change of the brain’s shape^42^. Additionally, gait improvement after the CSFTT in NPH patients was associated with an increase in regional cerebral blood flow in the middle gyrus of the frontal lobes and in the parahippocampal gyrus of the left temporal lobe^43^. As a result, given the chance of changes in brain state and subsequent changes in gait, we had an opportunity to validate that our vision-based gait analysis system maintained strong correlations with GAITRite for all parameters following CSFTT. The vision-based gait analysis method for temporospatial gait measurements may compare favorably with the GAITRite gait analysis system after the CSFTT in INPH.

Interestingly, our data showed that stride time and stride length variability measured by the vision-based gait analysis system were weakly but significantly correlated with the FAB score. Although the origin of the gait variability in INPH is not completely understood, our results suggest that gait variability measurements obtained with the vision-based system may reflect the performance on the FAB. As mentioned above, stride time and stride length variability measured by the GAITRite system were also correlated with FAB score^21^. However, the GAITRite system requires expensive equipment, dedicated laboratory space, and a trained technician. The vision-based system requires only a monocular camera and artificial intelligence algorithms. The low device cost, ease of use, and system portability make it accessible to most practitioners, and the ability to quantify gait parameters facilitates longitudinal analyses and comparisons of patients. Moreover, several findings in the literature support these results. In Alzheimer's disease, mean regional cerebral blood flow reduction in the prefrontal cortex was correlated with increased stride-to-stride variability^44,45^. In addition, some limited evidence in neurodegenerative diseases has suggested that the prefrontal cortex is associated with gait variability^45^. Our findings bear more connections to previous studies on INPH patients. For example, many previous studies on cerebral perfusion patterns in INPH patients point out a diffuse or frontal-dominant reduction in cerebral blood flow^13,14^; further, frontal hypoperfusion and frontal subcortical white matter disintegration have been associated with INPH symptoms including urinary incontinence and gait disturbance^13,46,47^. In addition, a previous study reported that the total FAB score was associated with brain single photon emission CT (SPECT) perfusion in the prefrontal cortex independently of gender, age, and MMSE^48^. The study suggested, moreover, that the FAB might be useful for evaluating diseases correlated with frontal dysfunction^48^. However, our results should be interpreted cautiously because of the relatively low statistical power and the limited number of participants. Further studies are needed to confirm these results.

Our study had a few limitations. The first limitation of this study is that gait variability analysis was based on a relatively small number of steps. We tried to overcome this obstacle by increasing the number of walking trials to four. However, it may be claimed that this is inadequate and longer walking distances are required to properly determine gait variability. A second limitation was that we included INPH patients regardless of responsiveness to the CSFTT in this study. However, the motivation for this was to follow the diagnostic criteria in the international INPH guidelines^22^. INPH is diagnosed on the basis of convergent evidence from the clinical history, physical examination, and brain imaging^22^. It was suggested that treatment responsiveness should not be used as the basis for diagnosis of INPH^22^. And gait evaluation is an essential part of the assessment of patients with INPH. However, following the Japanese guideline, clinical improvement after the CSFTT is an important indicator that enhances diagnostic certainty from possible to probable^3^. In addition, shunt surgery is indicated for patients with INPH who exhibit a positive CSFTT response^3^. Our findings encourage future studies with larger study populations, including both CSFTT responders and non-responders, and quantitative gait parameters measured by the vision-based gait analysis system to investigate the possibility of utilizing a quantitative gait analysis using deep learning algorithms on monocular videos as a neurophysiological biomarker to predict CSFTT response. A third limitation is that the CV values still show relatively weak agreement compared with the other mean gait parameters as shown in Table 2. The relative lower performance of CVs can originate from the accumulation of errors over two stages. The generated errors from the 1st stage should be propagated into the 2nd stage because of the sequential learning strategy. It can be argued that this problem can be alleviated by designing separated subnetworks for each type of gait parameter properly. For example, instead of two-staged training, we can compose two separated convolutional subnetworks in parallel, and based on the features extracted at each block, they can be used to estimate the means and CVs respectively. This structure can minimize mutual dependencies between different types of gait parameters. We leave this approach for our future works. A fourth limitation was that we did not analyze quantitative neuroimaging results in our INPH patients. Combining quantitative gait and neuroimaging investigations of INPH patients may help us determine those associations and potentially any underlying pathophysiological interrelationships. We also believe that there might be merit in utilizing a vision-based gait analysis method for temporo-spatial gait measurement in a relatively large sample of INPH patients.

In conclusion, the vision-based gait analysis system might provide comparable data to the GAITRite system when assessing gait dysfunction in INPH. The vision-based gait analysis system might also be in agreement with the GAITRite system for both stride time and stride length variability. Our findings suggest future studies are needed to investigate whether the vision-based system is useful for assessing the risk of falling in INPH patients. Further, the association between both stride time and stride length variability, as measured by the vision-based gait analysis method, and FAB scores suggests frontal lobe functions may be associated with gait variability measurements using the camera-based system for INPH patients.

## Data Availability

The datasets generated and analyzed during the current study are available from the corresponding author upon request.
